# Why Don't All Infants Have Bifidobacteria in Their Stool?

**DOI:** 10.3389/fmicb.2016.00834

**Published:** 2016-05-31

**Authors:** Gerald W. Tannock, Pheng Soon Lee, Khai Hong Wong, Blair Lawley

**Affiliations:** ^1^Department of Microbiology and Immunology, University of OtagoDunedin, New Zealand; ^2^Riddet Centre for Research Excellence, Massey UniversityPalmerston North, New Zealand; ^3^Mead Johnson NutritionSingapore, Singapore; ^4^Department of Human Nutrition, University of OtagoDunedin, New Zealand

**Keywords:** bifidobacteria, infants, fecal microbiota, Lachnospiraceae, child development

Members of the genus *Bifidobacterium* are abundant in the stool of most human infants during the initial exclusively milk-fed period of life, especially at an age of 2–3 months (Harmsen et al., 2000; Favier et al., 2002; Mariat et al., 2009; Coppa et al., 2011; Turroni et al., 2012; Yatsunenko et al., 2012; Tannock et al., 2013; Barrett et al., 2015). Bifidobacteria dominate the stool microbiota regardless of whether the infants are fed human milk or formula based on ruminant milk (cow or goat). However, bifidobacteria have about 20% higher relative abundances in human milk-fed compared to formula-fed babies (Tannock et al., 2013). The greater abundance of bifidobacteria in human-milk-fed infants can, at least in part, be explained by the fact that bifidobacterial species that are enriched in the infant bowel can utilize Human Milk Oligosaccharides (HMO) or their components as growth substrates (Sela et al., 2008; LoCascio et al., 2010; Garrido et al., 2013). It could be anticipated, therefore, that bifidobacteria would be detectable in the stool microbiota of every child nourished at the breast because of the supply of appropriate growth substrates. This expectation is not borne out completely because a proportion of infants have very low abundance or undetectable bifidobacteria as members of the fecal microbiota regardless of breast milk or formula feeding (Young et al., 2004; Gore et al., 2008; Tannock et al., 2013). Antibiotics had not been administered to these infants. How then can the absence of bifidobacteria be explained?

## A growth substrate deficit?

The “bifidobacteria-negative” babies have been detected in both human milk and formula-fed infants. Therefore, a bacterial growth substrate effect seems unlikely. While human milk is rich in HMO and ruminant milk lacks these complex molecules (although simpler forms such as sialylated-lactose are present in very small amounts), bifidobacteria are still the most abundant taxon in the feces of infants fed formula un-supplemented with galacto- or fructo-oligosaccharides (Tannock et al., 2013). In this case, lactose and/or glycoproteins and glycolipids are probable growth substrates for bifidobacteria (Turroni et al., 2010; Bottacini et al., 2014; O'Callaghan et al., 2015) in the bowel of exclusively milk fed infants. There is, however, a need to support genomic analysis of bifidobacteria with culture-based investigations of bifidobacterial nutrition based on substrates present in the bowel of exclusively milk-fed babies (other than HMO).

## Lack of sensitivity of bifidobacterial detection methods?

An obvious reason for bifidobacteria-negative feces is that the detection methods lack sufficient sensitivity. Culture-based methods usually have a lower detection limit of 1 × 10^3^ per gram, fluorescent in situ hybridization (FISH) 1 × 10^6^ − 10^7^ per gram (manual or digital counts respectively) or ~4 × 10^4^ by flow cytometry, and denaturing gradient gel electrophoresis of PCR amplicons ~1 × 10^5^ – 10^6^ cells (Welling et al., 1997; Jansen et al., 1999; Zoetendal et al., 2001, 2002) or 1 × 10^4^ using internal transcribed spacer targets (Milani et al., 2014). High throughput DNA sequencing methods, such as Illumina, generate tens of thousands of 16S rRNA gene sequences per DNA sample, but there may be several hundred OTU per sample. Thus, taxa present in very low abundance could be missed. However, reference to rarefaction curves (alpha diversity) during sequence analysis will show whether coverage of the microbiota is near complete or not. Therefore, while lack of sufficient sensitivity of detection methods remains a possibility, it probably does not provide the total explanation.

## Bifidobacterial populations rise and fall from day to day?

**Figure 1:**
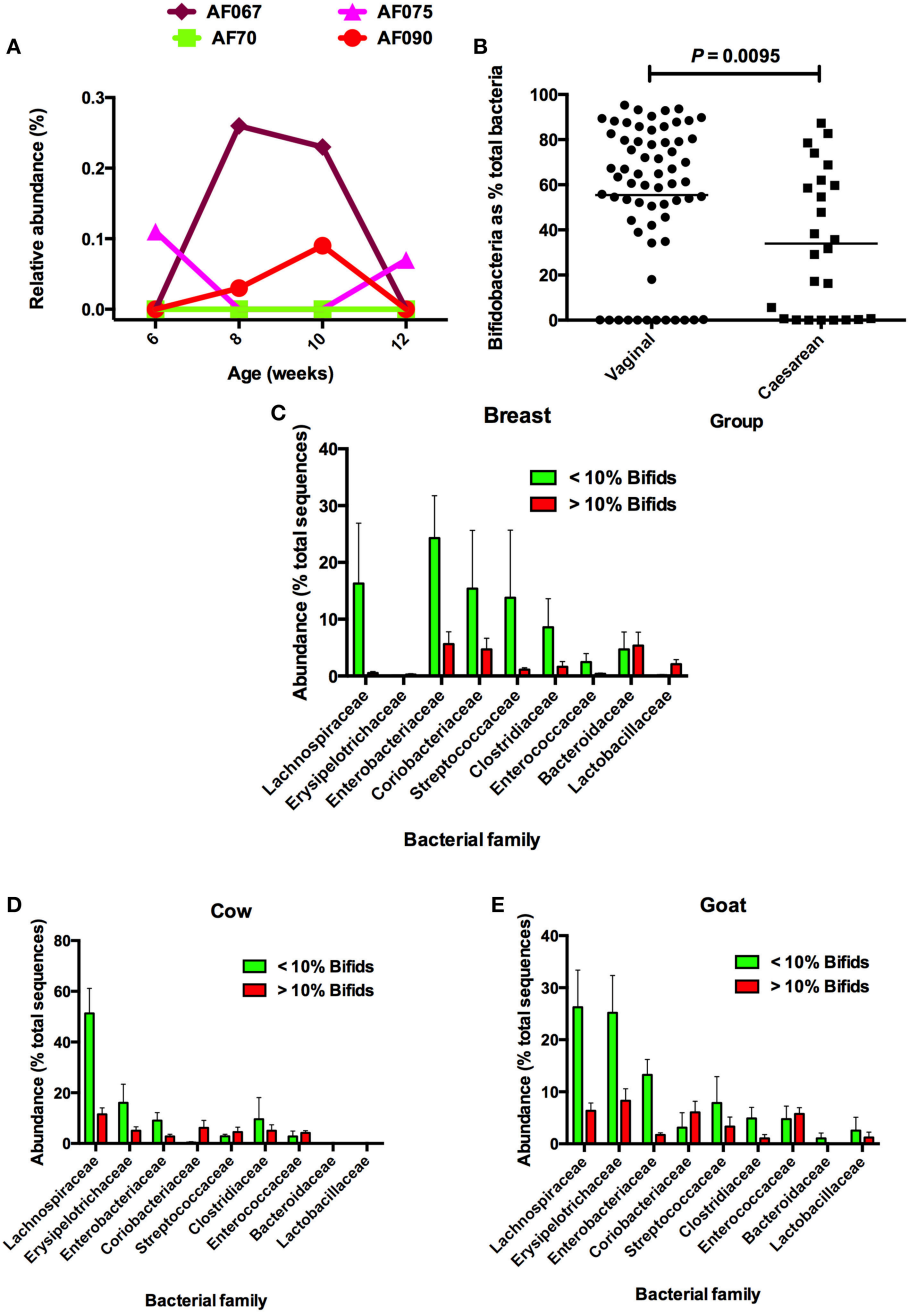
**(A)** Examples of babies without bifidobacteria, or very low abundances of bifidobacteria, in feces during the first 12 weeks of life. Bifidobacteria were undetectable by DNA sequencing of 16S rRNA gene amplicons at 6 and 12 weeks in the feces of AF067; 6, 8, 10, and 12 weeks in AF70; 8 and 10 weeks in AF075; and 6 and 12 weeks in AF090. **(B)** Comparison of bifidobacerial abundances in the feces of infants delivered vaginally or by cesarean. Note that in both groups, bifidobacteria were not detected in some infants. **(C)** Comparison of abundances of bacterial families in microbiotas of breast milk-fed infants in relation to abundances of bifidobacteria. **(D)** Comparison of abundances of bacterial families in microbiotas of cow milk formula-fed infants in relation to abundances of bifidobacteria. **(E)** Comparison of abundances of bacterial families in microbiotas of goat milk formula-fed infants in relation to abundances of bifidobacteria. Note that Lachnospiraceae have increased abundances when bifidobacteria have low relative abundance. Figures after Tannock et al. (2013), reproduced with permission.

Most fecal microbiota studies examine a single fecal sample from each participating individual. Comprehensive temporal studies of the fecal microbiota to determine day-to-day variations in composition have not been reported. It is possible that bifidobacteria are present in the feces of all children during early life but that, on some days, the bifidobacterial population falls to undetectable levels. Populations of bifidobacteria in the feces of some adults without diseases are dynamic in terms of strain composition, so there is some support for a concept of temporal instability in the bifidobacterial population of the microbiota (McCartney et al., 1996). Figure 1A shows data from feces collected at intervals from infants during the first 12 weeks of life. In the example, fluctuations in the abundances of bifidobacteria were seen, varying from very low abundance to absence, in feces of individual children. Strikingly, bifidobacteria were not detected in any of the fecal samples of one child. Therefore, bifidobacteria-free infants do seem to be a real phenomenon.

## The window of infectivity (opportunity/colonization) was missed?

A window of opportunity is a short time period during which an otherwise unattainable opportunity exists. After the window of opportunity closes, the opportunity ceases to exist. Caufield was the first to describe the “window of infectivity” in the acquisition of commensal bacteria. His example was *Streptococcus mutans* in the oral cavity of children (Caufield et al., 1993; Li and Caufield, 1995). This bacterial species is associated with dental plaque, thus the window of infectivity coincided with the eruption of the first molars. Prior to this, a habitat for *S. mutans* is not available in the oral cavity of children for this species. The Caufield hypothesis reminds us that many factors have to coincide to favor the establishment of a commensal in a body site. Cesarean-delivered babies have lower prevalences of bifidobacteria in their feces in early life (Figure 1B). By analogy to Caufield's studies, this probably relates to a lack of favorable opportunities for bifidobacteria to colonize the bowel relative to the vaginal birth process. Notably, we found that 36% of cesarean-derived babies lacked bifidobacteria, whereas 18% of vaginally delivered infants were bifidobacteria-free at 2 months of age (Tannock et al., 2013).

## Other taxa replace bifidobacteria in some babies?

If bifidobacteria have not colonized the bowel of certain infants, they are likely to be replaced by other taxa, which may have the requisite metabolic properties to fill the vacant ecological niche. In a study of the fecal microbiotas of Australian babies that were breast milk- or formula-fed, we compared the relative abundances of bacterial taxa in infants that had very low (< 10%) or higher (>10%) bifidobacterial content (Tannock et al., 2013). Analysis of the compositions of these microbiotas showed that when *Bifidobacteriaceae* abundance was low, *Lachnospiraceae* abundances tended to be greater in babies in all dietary groups (Figures 1C–E). There was also a tendency for *Erysipelotrichaceae* abundances to be greater in formula-fed babies with low bifidobacterial abundances, being much more evident in the case of goat milk-fed infants. These observations suggest that, yes, other taxa might replace bifidobacteria in the fecal microbiota of some children.

## What are the consequences of lacking bifidobacteria in the bowel?

The absence of bifidobacteria in the bowel may be detrimental for infant development. The curious phenomenon whereby mother's milk contains substances not used in the nutrition of the offspring, but which fertilize bifidobacterial growth, is unique to humans. There must be a good reason for this. Enriching bifidobacterial populations in the bowel tends to minimize the abundance of other bacterial species, so a competitive exclusion function could be ascribed to HMO. Additionally, HMO may act as “decoys” in the bowel by binding to pathogens (bacteria and viruses) and their toxins and thus limiting contact with mucosal surfaces (Kunz et al., 2000). The large diversity of HMO structures that is known to occur in human milk suggests a large diversity of decoy functions (Pacheco et al., 2015). Irrespective of where in the World babies live, their gut microbiomes are enriched in genes involved in the *de novo* biosynthesis of folate (Yatsunenko et al., 2012). In contrast, the microbiome of adults favors synthesis of another B vitamin, cobalamin. Folate synthesis is an attribute of bifidobacteria and folate can be absorbed from the large bowel, so enrichment of bifidobacteria in the infant bowel may provide an important contribution to infant nutrition (Aufreiter et al., 2009; D'Aimmo et al., 2012; Lakoff et al., 2014). Folate functions as a coenzyme or co-substrate in single-carbon transfers in the synthesis of nucleic acids and metabolism of amino acids. One of the most important folate-dependent reactions is the conversion of homocysteine to methionine in the synthesis of S-adenosyl-methionine, an important methyl donor. Another folate-dependent reaction, the methylation of deoxyuridylate to thymidylate in the formation of DNA, is required for proper cell division (Crider et al., 2012). Neonatal nutrition could, indeed, be the very important reason for the HMO-bifidobacteria-infant paradigm. The foundation of brain structure and function is set early in life through genetic, biological and psychosocial influences. The rate of neonatal brain growth exceeds that of any other organ or body tissue (Wang, 2012). The infant is born with neurons already formed but the synaptic connections between these cells are mostly established and elaborated after birth causing a large nutritional demand for biosynthesis of gangliosides (Svennerholm et al., 1989). Nutrition of the infant in early life affects brain developmental processes including cognition (Uauy and Peirano, 1999; Uauy et al., 2001). While long-chain fatty acids (such as docosahexaenoic acid) have been the focus of much of the research in this field, tantalizing research evidence now indicates that sialic acid (N-acetyl-neuraminic acid), a 9-carbon carbohydrate, is also an essential nutrient for optimal brain development and cognition (Gibson, 1999; Meldrum et al., 2012; Wang, 2012). Strikingly, cortical tissue from human brain contains up to 4 times more sialic acid than that of other mammals tested (Wang et al., 1998). Moreover, the sialic acid concentration in the brain of breast milk-fed babies is higher than in that of formula-fed infants (Wang et al., 2003). These facts correlate with the unique biochemistry of human milk and the unique bacteriology of the infant bowel. Intriguingly, Ruhaak et al. (2014) have reported the detection of sialylated oligosaccharides (3′ sialyl-lactose, 6′ sialyl-lactose, 3′ sialyl-lactosamine, 6′ sialyl-lactosamine) that might result from the hydrolysis of HMO, in the blood of human infants. Thus, bifidobacterial biochemistry in the bowel may have extra-intestinal, nutritional influences important in brain development. However, perhaps the taxa that are abundant in the bowel of infants in the absence of bifidobacteria can carry out these same functions? This interesting possibility remains to be investigated.

## Babies without bifidobacteria are important sources of knowledge?

Rene Dubos explored in a number of books the interplay between environmental forces and the physical, mental, and spiritual development of humankind. His article published in the journal *Pediatrics* entitled “Biological Freudianism: lasting effects of early environmental influences” encapsulated this theme (Dubos et al., 1966). Drawing on the results of experiments conducted with specific-pathogen-free mice, the authors concluded that “From all points of view, the child is truly the father of the man, and for this reason we need to develop an experimental science that might be called biological Freudianism. Socially and individually the response of human beings to the conditions of the present is always conditioned by the biological remembrance of things past.”

Biological Freudianism is clearly of relevance to the concept that the first 1000 days, between conception and the child's second birthday, offer a unique window of opportunity to shape healthier and more prosperous futures. Nutrition during this 1000 day window can have a profound impact on a child's ability to grow, and learn. The influences of the microbiota on the development of the child during early life are potentially very important, and much longitudinal research is required to clarify whether there are continuing, medically important impacts of the microbiota, inlcuding the bifidobacteria, that last throughout the lifetime of humans. Comparisons of the cognitive development and general health status of children that had been bifidobacteria-free, and children that were ex-bifidobacteria-free then intentionally exposed to bifidobacteria, in a longitudinal study extending perhaps 10 or 20 years, would tell us whether these bacteria optimize short and/or long term human development and health.

## Author contributions

GT wrote the article. BL, PL, and KW provided data described in the article.

### Conflict of interest statement

The authors declare that the research was conducted in the absence of any commercial or financial relationships that could be construed as a potential conflict of interest.
